# Protocol for the development and validation procedure of the managing the link and strengthening transition from child to adult mental health care (MILESTONE) suite of measures

**DOI:** 10.1186/s12887-020-02079-9

**Published:** 2020-04-16

**Authors:** P. Santosh, L. Adams, F. Fiori, N. Davidović, G. de Girolamo, G. C. Dieleman, T. Franić, N. Heaney, K. Lievesley, J. Madan, A. Maras, M. Mastroianni, F. McNicholas, M. Paul, D. Purper-Ouakil, I. Sagar-Ouriaghli, U. Schulze, G. Signorini, C. Street, P. Tah, S. Tremmery, H. Tuomainen, F. C. Verhulst, J. Warwick, D. Wolke, J. Singh, S. P. Singh, for the MILESTONE Consortium

**Affiliations:** 1 Department of Child and Adolescent Psychiatry, King’s College London, London, UK; 2 Centre for Interventional Paediatric Psychopharmacology and Rare Diseases, South London and Maudsley NHS Foundation Trust, London, UK; 3 HealthTracker Ltd, Gillingham, Kent, UK; 4 School of Psychology, Plymouth University, Plymouth, UK; 5 Department of Psychiatry, Clinical Hospital Center Split, Split, Croatia; 6 Unità di Psichiatria Epidemiologica e Valutativa, IRCCS Istituto Centro San Giovanni di Dio Fatebenefratelli, Brescia, Italy; 7 Department of Child and Adolescent Psychiatry and Psychology, Erasmus Medical Center, Rotterdam, The Netherlands; 8 Warwick Clinical Trials Unit, Warwick Medical School, Warwick Medical School, Coventry, UK; 9 Yulius Academy, Rotterdam, The Netherlands; 10 Department of Child and Adolescent Psychiatry and Psychology, Erasmus Medical Center, Rotterdam, The Netherlands; 11 Department of Child and Adolescent Psychiatry, University College Dublin School of Medicine and Medical Science, Dublin, Republic of Ireland; 12 Geary Institute, University College Dublin, Dublin, Republic of Ireland; 13 Department of Child Psychiatry, Our Lady’s Hospital for Sick Children, Dublin, Republic of Ireland; 14 Lucena Clinic, SJOG, Dublin, Republic of Ireland; 15 Centre for Mental Health and Wellbeing Research, Warwick Medical School, University of Warwick, Coventry, UK; 16 Coventry and Warwickshire Partnership NHS Trust, Coventry, UK; 17 CHU Montpellier / University of Montpellier; Saint Eloi Hospital, Médecine Psychlogique de l’enfant et de adolescent (MPEA1), Montpellier, France; 18 Department of Child and Adolescent Psychiatry/Psychotherapy, University of Ulm, Ulm, Germany; 19 Department of Neurosciences, Child & Adolescent Psychiatry, University of Leuven, Leuven, Belgium; 20 Department of Child & Adolescent Psychiatry, University Hospitals Leuven, Leuven, Belgium; 21 Department of Clinical Medicine, University of Copenhagen, Copenhagen, Denmark; 22 Department of Psychology, University of Warwick, Coventry, UK

**Keywords:** Adult mental health services, Child and adolescent mental health services, Europe, Patient reported outcome measures, Transition, Young persons

## Abstract

**Background:**

Mental health disorders in the child and adolescent population are a pressing public health concern. Despite the high prevalence of psychopathology in this vulnerable population, the transition from Child and Adolescent Mental Health Services (CAMHS) to Adult Mental Health Services (AMHS) has many obstacles such as deficiencies in planning, organisational readiness and policy gaps. All these factors contribute to an inadequate and suboptimal transition process. A suite of measures is required that would allow young people to be assessed in a structured and standardised way to determine the on-going need for care and to improve communication across clinicians at CAMHS and AMHS. This will have the potential to reduce the overall health economic burden and could also improve the quality of life for patients travelling across the transition boundary. The MILESTONE (Managing the Link and Strengthening Transition from Child to Adult Mental Health Care) project aims to address the significant socioeconomic and societal challenge related to the transition process. This protocol paper describes the development of two MILESTONE transition-related measures: The Transition Readiness and Appropriateness Measure (TRAM), designed to be a decision-making aide for clinicians, and the Transition Related Outcome Measure (TROM), for examining the outcome of transition.

**Methods:**

The TRAM and TROM have been developed and were validated following the US FDA Guidance for Patient-reported Outcome Measures which follows an incremental stepwise framework. The study gathers information from service users, parents, families and mental health care professionals who have experience working with young people undergoing the transition process from eight European countries.

**Discussion:**

There is an urgent need for comprehensive measures that can assess transition across the CAMHS/AMHS boundary. This study protocol describes the process of development of two new transition measures: the TRAM and TROM. The TRAM has the potential to nurture better transitions as the findings can be summarised and provided to clinicians as a clinician-decision making support tool for identifying cases who need to transition and the TROM can be used to examine the outcomes of the transition process.

**Trial registration:**

MILESTONE study registration: ISRCTN83240263 Registered 23-July-2015 - ClinicalTrials.gov NCT03013595 Registered 6 January 2017.

## Background

In the coming decade, the burden of mental ill health in children and young people is expected to increase by 50% [1]. A transition from childhood to adulthood can open new opportunities for young people; however, it can also be a period of emotional and physical challenges. For those who are mentally ill, this journey can be daunting especially when faced with the transition from a child and adolescent mental health services (CAMHS) to adult mental health services (AMHS). The transition boundary represents a precarious point at a critical life stage for young people and is beset with inadequate provision of care [2, 3]. Failure of care at this transition boundary due to disengagement of services [4, 5] can have a significant impact on young people and their subsequent quality of life and contribution to society [6], for example, conditions that were relatively straightforward to treat in their early stages becoming entrenched with adverse social, employment and housing implications. The transitions of Care from Child and Adolescent Mental Health Services to Adult Mental Health Services (TRACK) [7–9] study also showed that the majority of young people with mental health needs not referred from CAMHS to AMHS had emotional disorders or neurodevelopmental disorders [10], suggesting that young people with these conditions are at most risk of being failed by healthcare services/falling through the gap.

Despite transition being highlighted by the Department of Health in the National Health Service, England (NHS, England) [11] and the National Institute for Health and Care Excellence [12] as a key area needing improvement, there is paucity in the evidence base relating to models that aim to improve care at the transition boundary between mental health services. There is also a lack of shared decision making across different countries [13] that further hampers the development of such models. In other geographical regions such as in Canada, others have shown that engagement methods using digital approaches i.e., ‘Thought Spot’, a web-based platform that aims to facilitate transition in youth, can be useful for those in post-secondary settings wishing to access mental health services [14]. More recently, another study is tracking experiences of young people in CAMHS as they transition through the CAMHS/AMHS boundary [15]. This Longitudinal Youth in Transition Study (LYiTS) is important because it would be the first prospective longitudinal study to assess transition in North America and together with the findings from the MILESTONE study would provide important information for young people in mental health care as they transition across the transition boundary. Other work has explored strategies to improve the transition of marginalized youth into adulthood [16]. This study identified themes that could assist in the transition process. Some important themes raised in the context of transition were the engagement of involved parties to improve service delivery, the impact of relationships with adults and engagement with family members.

The need for a robust, standardised model of transition has been expressed [17–19], especially one which incorporates an evidence-based decision-making process for identifying those young people who should make a transition to AMHS, those who can be managed by other services [20, 21], and those who could be discharged from CAMHS. Another model has focused on other factors that could improve the mental health of young people such as the OnTrackNY, that aims to provide early intervention services for young people experiencing psychosis [22, 23] while others have looked to address the elements involved in youth seeking treatment for substance abuse [24]. The Transition to Independence Process model also addressed factors for transition aged youth [25]. Taken together these findings highlight that transition for young people is complex and several elements need to be considered. Across Europe and other regions, there are indications that service provision at the transition boundary is precarious and would benefit from the development of such a needs-based assessment [13, 26, 27]. Empowering clinicians with information from an accurate measure focusing on relevant domains will enable a smoother and purposeful process of transition from CAMHS to AMHS or discharge from mental health services if there is no longer a clinical need.

### Current transition-related measures

Measures to evaluate the preparedness of transition have been examined in young people and adults with chronic and special healthcare needs [28, 29], however, other measures to test the effectiveness of transition in community settings are scarce. Only a few scales have addressed transitions within mental health services. One study explored the readiness for the transition of treatment into the community and cited domains such as housing, treatment engagement, medication use, high-risk behaviours and substance abuse to help manage assertive community treatment (ACT) team capacity [30]. Another such as the University of North Carolina (UNC) TR(x)ANSITION Scale [31], is a disease-neutral tool that can be employed in the clinic to measure the components of paediatric health care transition to adult care. This scale has items that are deemed necessary for the transition process such as self-management, medication adherence, knowledge of the condition, navigation of services, social support and community involvement. Through interviews with hospitalised, chronically ill adolescents, another study focused on independence including attendance of hospital appointments and coping with the condition [32]. Many of the items in this scale were related to the ‘readiness for transition’ rather than the appropriateness for transition amongst mental health populations. Other measures include the continuity of care in mental health services measure, CONNECT [33], the Patient Continuity of Care Questionnaire [34] and the Alberta Continuity of Services Scale - Mental Health (ACSS-MH) [35]. There are other measures of transition, yet few of them relate to the mental health care setting. A detailed systematic review on measures of readiness to transition excluded ones that specifically targeted mental health or developmental disorders [36]. Another review focused on transition outcomes on mental health [37] but revealed a scarcity of studies with sufficient power, precluding the drawing of any inferences on the effectiveness of different transition measures.

More recently, in the UK, an intervention has been developed as a co-production with young people (*n* = 18) who had experienced transition or were undergoing transition [38]. In this study, the anxiety of CAMHS leavers was underestimated by mental health services, and most young people viewed the CAMHS transition process as uncaring, feeling uninvolved or not being adequately informed of the transition process. These findings underscore the requirement for robust and comprehensive measures that can assess transition across the CAMHS/AMHS boundary.

### Aim

The MILESTONE (Managing the Link and Strengthening Transition from Child to Adult Mental Health Care) project aims to address the significant socioeconomic and societal challenges related to transition, in part by developing two transition-related measures for the MILESTONE study [39]. This paper describes the methodology linked with the development and validation of these bespoke MILESTONE measures related to transitioning from CAMHS: 1) the Transition Readiness and Appropriateness Measure (TRAM) for determining readiness and appropriateness for transition; 2) the Transition Related Outcome Measure (TROM) for examining the outcome of transition. The measures are holistic in both their scope and the process of development, to ensure that the young person is seen as more than a list of symptoms and involves not only clinicians but also young people and their parents/carers.

## Methods

**Fig. 1:**
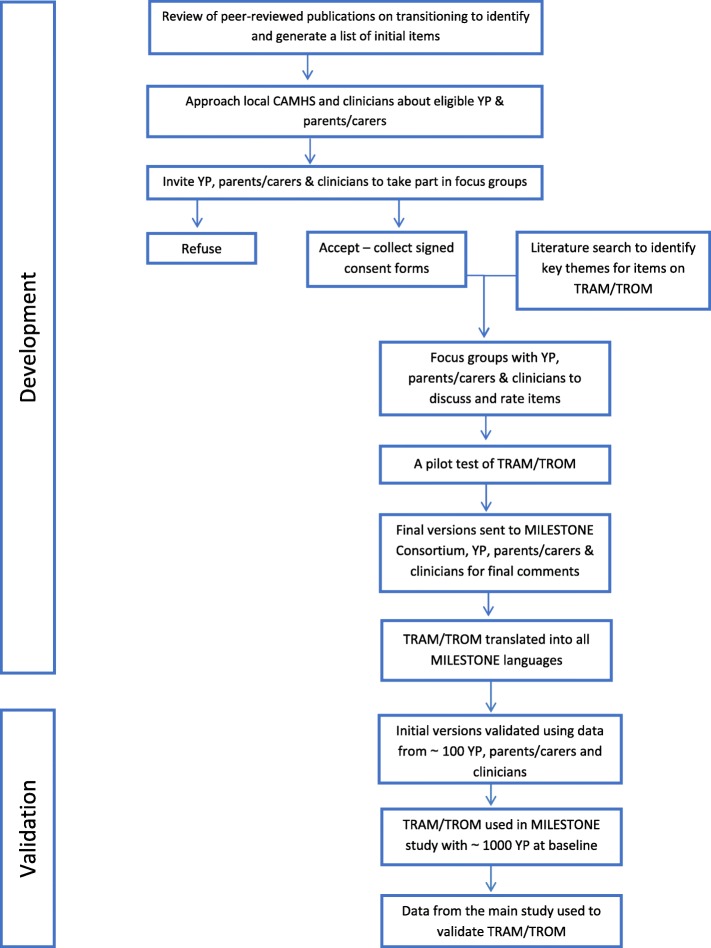
Flowchart for TRAM/TROM development and validation. Abbreviations: ~ (approximately); CAMHS (Child and Adolescent Mental Health Services); MILESTONE (Managing the Link and Strengthening Transition from Child to Adult Mental Health Care); TRAM (Transition Readiness and Appropriateness Measure); TROM (Transition Related Outcome Measure); YP (Young Person)

The TRAM and TROM measures were developed (see Fig. 1) as per guidelines described in the United States (US) Food and Drug Administration (FDA) Guidance for Industry Patient-Reported Outcome Measures (US FDA, 2009) [40]. These guidelines have previously been used for the development of measures in a rare disease population [41] and to assess mental health in individuals with autism [42]. This process is stepwise involving seven stages: 1) literature review, 2) review of items by experts, 3) focus groups, 4) production of draft scales, 5) scale testing and revision, 6) translation of scales, and 7) scale validation. The evaluation of the psychometric properties was a two-stage process: content validity, construct validity and test-retest reliability was assessed first using data from approximately 100 participants of the MILESTONE validation study (Phase 1, MILESTONE validation study) and responsiveness and interpretability were assessed subsequently using separate data from the main MILESTONE study (MILESTONE cohort study and nested cluster randomised trial). All stages of the MILESTONE study have been completed.

### STAGE 1: literature review for concept identification and concept elicitation

A search of peer-reviewed publications on transitioning between mental health services and continuity of care were used to generate an initial list of items that are deemed to be of importance by relevant experts in the field when considering the transition in a mental health context. These members were chosen due to their extensive experience of working in and knowledge of transitioning in CAMHS. Based on the literature review, members of the MILESTONE Consortium also discussed whether the use of one scale or multiple sub-scales was appropriate. The free solicitation of information during initial focus groups (see next section) also contributed to concept/item generation.

### STAGE 2: review of items by an international expert panel

Psychologists and psychiatrists from health institutions across the European Union (EU) (including members of the MILESTONE Consortium), with experience of transition, were recruited as an expert panel to provide feedback on the original list of scale items. Members of the MILESTONE consortia helped with a ranking of the long list and provided feedback on later versions of the scale.

### STAGE 3: focus groups

Young people with experience of CAMHS both pre and post-transition and their parents and carers were recruited to take part in focus groups and pilot testing of the measures.

#### Inclusion criteria for focus groups

Young people aged 16 to 19 years who had experience of working with CAMHS, had no intellectual impairment (IQ > 70), and had a reasonable fluency in the English language were eligible. Any parent/carer of a young person with experience of CAMHS was also able to participate providing they have an IQ above 70 and sufficient English to contribute to a discussion. Parents were able to participate without their child also participating. Mental health professionals were eligible to participate providing they have worked in a service for young people with mental health problems; this could be in a CAMHS service, an AMHS that accepts referrals from CAMHS or a community organisation.

#### Exclusion criteria for focus groups

Young people were deemed ineligible if they were under 16 years old, had an intellectual impairment (IQ < 70) or were considered to be too unwell to participate. If the participant was not able to (or was expected not to be able to) complete the questionnaires due to severe physical disabilities, even with assistance from family members or a research assistant, or deemed to be too vulnerable by their clinician, then he/she was not eligible. Furthermore, if participants did not have a reasonable level of English they were excluded from the study because a reasonable level of English was required to discuss the elements of transition and complete transition-related measures.

#### Participant selection

The three participant types (patients [young people], parents/carers and clinicians that have experience of transition) were recruited through mental health services, community organisations and advisory groups in London and Coventry & Warwickshire using convenience sampling. Clinicians who were known to the research teams and other clinicians in the selected organisations were approached to help with recruitment. They checked young people’s and their parents’/carers’ interest in participation after which a member of the research team contacted them. Study posters were also displayed in relevant clinic areas.

Information sheets and consent forms were provided to potential participants, with emphasis that participation is entirely voluntary. A minimum of 24 h was given between the provision of information and the actual recruitment of participants, who were asked to sign consent forms. Young people and their parents/carers were compensated for their time with a £10 high street shopping voucher.

Up to 100 participants, comprising young people with experience of CAMHS, their families and mental health care professionals, were involved in focus groups and pilot testing of the new scales. These participants were involved in the initial part of the focus groups and test-re-tested the measures.

#### Process of focus groups

In the context of NHS England, an NHS Trust is an organisation in the NHS that involves and engages with service users, patients, public and staff and resides in a particular geographical area. In the context of this study, focus groups took place at South London and Maudsley NHS Foundation (SLaM) Trust and Coventry and Warwickshire Partnership NHS Trust (CWPT). In each Trust, focus groups were held for young people, parents/carers, CAMHS clinicians and AMHS clinicians with each group comprising of only one participant type and a maximum of five participants. Nine (9) focus groups were held, the sessions were also audio-recorded, and detailed notes were made.

Initially, the focus groups centred on two themes (I) ‘readiness for transition’ and (II) ‘identifying successful transition’. The purpose of this discussion was to provide members of the research team with an idea of what the scales should be able to capture. Open-ended questions were used to ascertain the factors that participants consider to be important when determining whether a young person should transition from CAMHS to AMHS. Next, participants were provided with the initial list of items generated from the literature (and revised in subsequent focus groups) to rank them on a scale of 1–10, with 1 being unimportant and 10 being very important when deciding on transition. This same list of items was also presented to the international expert panel of mental health clinicians with experience in service user transition from CAMHS.

#### Analysis of focus groups

All focus groups were audio recorded and transcribed. A member of the research team throughout took notes. The transcripts allowed a rapid analysis of the data before the next focus group. After each focus group, the results of the importance ratings for each item was analysed, and any new items generated was added to the list. Potential scale items relating to transition appropriateness and transition outcome were defined based on consensus agreement with the expert panel from the MILESTONE consortium.

### STAGES 4 & 5: production of draft scales and scale testing and revision

Next, initial versions of the TRAM and TROM were developed and discussed, and pilot tested with participants in further focus groups. Unstructured qualitative interviews were conducted to identify wording and completion problems and to gain feedback on the inclusion of items. Members of the MILESTONE Consortium also held in-depth discussions on the most appropriate patterns of response and measurement options, i.e., Likert style scales, detailed checklists and standalone items, and on minimising completion burden. Comments on the clarity and readability of written item descriptions were solicited at all focus group sessions. Scale templates (wireframes) were also presented during the focus groups to see how participants would like the TRAM and TROM scales to appear on the web-based HealthTracker™ platform. Modification and re-evaluation of measures were conducted based on feedback. Scales were sent to the MILESTONE consortia members to check for issues with the scales such as including items that do not translate between languages. Lastly, scales containing the final items were also sent to clinicians, parents, young person advisors and young people for comment upon usability, content, and structure.

### STAGE 6: translation of scales

Final versions of the scales were then translated from English into all MILESTONE languages (French, French [for Belgium], German, Dutch, Dutch [for Belgium], Croatian and Italian). The process involved translation, back translation and back translation review by the team who created the scale. Any inconsistencies detected in the back-translation review were discussed and amended to ensure that meanings were consistent across language versions.

Both scales were developed with versions for young people, clinicians and parent/carers with similar questions asked from all participant types. The aim was for all included items to be stand-alone as they appear singly when the final version of the scale is displayed digitally. This was recommended by the MILESTONE consortium that consisted of an expert panel of psychologists and psychiatrists from health institutions across the EU, with knowledge and experience of transition. As far as possible, all items are worded simply and concisely and rated over a similar period (e.g., 6 months).

#### Web-based presentation of the TRAM and TROM on the HealthTracker™ platform

The web-based health monitoring platform HealthTracker™ has been used successfully in other multi-centric studies [42, 43]. The TRAM and TROM were designed as user-friendly online assessments that exploited the functionality of the web-based HealthTracker™ platform, allowing the measures to be completed remotely using developmentally appropriate interfaces, branching structure of questions, and allocation of appropriate questionnaires based on need and time-point in the study. They formed part of the MILESTONE study assessment package [39].

### STAGE 7: scale validation

The validation process of the MILESTONE measures has been completed. This process was done to ensure that the developed measures assessed the parameters that they were designed to (validity) and that they did this consistently (reliability). Additionally, the validation assisted in improving accuracy, accessibility and minimising completion burden. All three versions (young person [YP], parent/carer [PC] and clinician [CL]) of the TRAM and TROM were validated in all the MILESTONE Consortium languages. There were two phases to the validation: phase 1 (MILESTONE validation study) assessed content validity, construct validity and phase 2 (MILESTONE cohort study and nested cluster randomised trial) assessed responsiveness and interpretability, and the psychometric properties. For the first phase, a pilot study was conducted in the eight MILESTONE countries (United Kingdom, France, Italy, Netherlands, Germany, Belgium, Ireland, and Croatia), with further details below.

### Sample size

For the preliminary validation of the scales, the total sample size across the eight countries was calculated to be approximately 100 participants in each group (i.e., 100 young people, 100 parents/carers/spouses, and 100 mental health professionals), which was based on sample size calculations.

The power calculations linked with the external validation have been described in the protocol paper for the MILESTONE study [39]. For the analysis of external validity, all participants in the MILESTONE study (the cohort and control arms) participated, resulting in a group of approximately 3000 participants (1000 YP, 1000 PC and 1000 CL).

### Recruitment targets

For the first phase of validation, each participant country was to recruit 15 young people alongside 15 parents/carers/spouses and 15 mental health care professionals; from these 15-young people, at least 10 should have transitioned from CAMHS to AMHS within 18 months. The remaining five participants could be from either group.

For the second phase of validation, different inclusion targets for young people (and associated parents/carers and clinicians) were set for each participant country, depending on the number of CAMHS clusters included in the MILESTONE study [39].

### Analyses plan

Quantitative data is being analysed using the latest version of the SPSS statistical package (IBM SPSS Statistics for Windows. Armonk, NY: IBM Corp.).

### Phase 1 of validation

#### Content validity

The content validity of the TRAM and TROM was assessed to see whether the items and response options are relevant measures of the construct.

#### Criterion validity

The discriminative power (validity) of both scales were assessed. The primary outcome measure of the MILESTONE study is health status as measured by Health of the Nation Outcome Scale for Children and Adolescents (HoNOSCA) [44] whose content validity has been established [45, 46]. For this study, the newly developed scales were compared against other standard scales such as the HoNOSCA (self-rate and clinician-rated versions) measure as well as other scales namely the Clinical Global Impression-Severity (CGI-S) and improvement (CGI-I) scales [47] using the Pearson’s product moment correlation coefficient. The specific subscale scores of the developed transition scales were also analyzed using Pearson’s correlation coefficients to see whether they correlate to the Specific Levels of Functioning (SLOF) scale (parent-rated).

#### Internal consistency

Cronbach’s alpha for summary scores were calculated for the TROM and TRAM. Alpha (α) values of 0.80 or higher are commonly accepted as evidence of adequate internal consistency [48]. If relevant, ‘alpha if deleted analyses’ was also performed to see if removing any potential item(s) from the scales, would reinforce the measures.

#### Test-retest reliability

The correlation coefficients between Timepoint 0 (TP0) (first completion) and TP1a (the second completion was done within ≤41 days of first assessment) were calculated using ANOVA. Inter-rater reliability of scales from different raters at the respective time points was computed (the second completion was done within ≤41 days of first assessment [TP1a]).

### Phase 2 of validation

#### Responsiveness and interpretability

The responsiveness and interpretability of the TRAM and TROM were assessed using data obtained from the main MILESTONE study, with a total of approximately 1000 young people and associated parents/carers and CAMHS and AMHS clinicians recruited at baseline [39] after data collection for the main study had been completed. Statistical analysis was done to obtain a final factor structure, sensitivity, specificity and predictive value of the TRAM and TROM. Exploratory factor analyses (EFA) (principal axis, Promax rotation) was performed on the different versions of the TROM and TRAM subscales.

After analysing the data, the scales were optimised, by checking to see if any items could be dropped from the scales to make them simpler. The predictive validity of TRAM was also assessed, by performing statistical analyses to identify discriminators of successful and unsuccessful transition, and a MILESTONE Transition Predictor was developed from the final version of TRAM. This transition predictor is formatted similar to a traffic light scoring system and allows the development of future analytics to look at data across all time points at the end of the study and whether the outcomes of transition can be predicted based on symptom profile.

## Discussion

This study protocol reports the development of two transition related measures: the TRAM and TROM. These measures are web-based measures on the HealthTracker™ platform and were translated into eight European languages and are being tested in eight EU countries in a two-phase process. The first phase involved approximately 100 young people and covered construct validity, content validity, and test-retest validity. The second phase involved over 1000 young people to test responsiveness and interpretability. The development and validation of the TRAM and TROM has been completed.

As the HealthTracker™ based TRAM and TROM measures are web-based, they have the potential to be used worldwide by end users thereby contributing to a smoother transition process and allowing for personalised mental health care and have added value in informing the transition process from CAMHS to AMHS. The findings from these measures will be presented in meetings and conferences and published in scientific journals. A MILESTONE specific website has already been established to facilitate dissemination activities (http://milestone-transitionstudy.eu).

A potential limitation of this study is that the study focuses on a population which is difficult to recruit (adolescent mental health service users). Furthermore, participants with the poorest health may be least likely to respond, or most likely to have missing data.

In summary, the TRAM and TROM measures are novel in the sense that they can be provided to clinicians as a decision-making support tool to identify cases that need to transition and the outcomes of it. This will increase our understanding of the transition process.

## Data Availability

Not applicable.

## Abbreviations

AMHS: Adult Mental Health Services
CAMHS: Child and Adolescent Mental Health Services
CL: Clinician
EU: European Union
MILESTONE: Managing the Link and Strengthening Transition from Child to Adult Mental Health Care
NHS: National Health Service
PC: Parent/carer
TRAM: Transition Readiness and Appropriateness Measure
TROM: Transition Related Outcome Measure
YP: Young Person
